# Characteristics of Multisystem Inflammatory Syndrome in Children Across COVID-19 Variants in Vojvodina

**DOI:** 10.3390/jcm13226672

**Published:** 2024-11-06

**Authors:** Gordana Vijatov-Đurić, Borko Milanović, Nenad Barišić, Jelena Ivetić, Andrea Đuretić, Jelena Kesić, Ognjen Ležakov, Ivana Vorgučin, Gordana Vilotijević-Dautović, Mioljub Ristić, Katarina Koprivšek, Vesna Stojanović

**Affiliations:** 1 Faculty of Medicine, University of Novi Sad, 21000 Novi Sad, Serbia; gordana.vijatov-djuric@mf.uns.ac.rs (G.V.-Đ.); borko.milanovic@mf.uns.ac.rs (B.M.); andrea.djuretic@izzzdiovns.rs (A.Đ.); jelena.kesic@mf.uns.ac.rs (J.K.); ivana.vorgucin@mf.uns.ac.rs (I.V.); gordana.vilotijevic-dautovic@mf.uns.ac.rs (G.V.-D.); mioljub.ristic@mf.uns.ac.rs (M.R.); katarina.koprivsek@mf.uns.ac.rs (K.K.); vesna.stojanovic@mf.uns.ac.rs (V.S.); 2 Institute for Child and Youth Healthcare of Vojvodina, 21000 Novi Sad, Serbia; 3 Faculty of Technical Sciences, Univerity of Novi Sad, 21000 Novi Sad, Serbia; jelenaivetic@uns.ac.rs; 4 Institute of Public Health of Vojvodina, Department of Epidemiology, 21000 Novi Sad, Serbia

**Keywords:** COVID-19, MIS-C

## Abstract

**Background/Objectives**: To investigate if the severity and presentation of multisystem inflammatory syndrome in children (MIS-C) vary between different severe acute respiratory syndrome coronavirus 2 (SARS-CoV-2) variants. **Methods**: This retrospective study included 59 patients aged 0–18 years, diagnosed with COVID-19 and MIS-C, treated and monitored over a one-year period after discharge from hospital. The patients were grouped according to the predominant SARS-CoV-2 variant. The predominant variant of SARS-CoV-2 was assumed by the date of hospitalization. The following patient data were collected: demographic data (age, sex), information on comorbidities, body mass index, clinical data (fever and duration of febrile periods, symptoms of Kawasaki-like phenotypes, and presence of respiratory, cardiovascular, gastrointestinal, neurological and other symptoms), and laboratory and imaging findings. **Results**: In total, 24 (41%), 19 (32%), and 15 (25%) patients were diagnosed with MIS-C during the Alpha, Delta, and Omicron periods, respectively (63.8% were males; 36.2% were females). Comorbidities were present in 49% of patients. Respiratory symptoms were the most common during the Delta period (73%, *p* = 0.028). There was no statistically significant difference in the occurrence of other symptoms, laboratory findings, treatment, complications, and long-term outcomes between groups. **Conclusions**: No significant correlation was found between hospitalization date (used to estimate COVID-19 variant) and presentation/severity of MIS-C.

## 1. Introduction

After December 2019, when the first coronavirus disease 2019 (COVID-19) cases associated with severe acute respiratory syndrome coronavirus 2 (SARS-CoV-2) were registered in China, the disease rapidly expanded, resulting in a global pandemic [1]. Contrary to the adult population, children mainly manifested mild disease symptoms [2]. However, in April 2020, first in the United Kingdom and Italy, and afterward in other European regions and the USA, clinical syndromes resembling Kawasaki disease or toxic shock syndrome were reported, which occurred after the preceding SARS-CoV-2 infection. The condition has been termed multisystem inflammatory syndrome in children (MIS-C) [3,4,5]. MIS-C is a rare but severe complication of COVID-19 that occurs in approximately 1 in 3000 children and typically develops 2–6 weeks after commonly asymptomatic and mild SARS-CoV-2 infection. Symptoms of MIS-C include fever, rash, conjunctival injection, abdominal pain, vomiting/diarrhea, shock, etc. Several cases of MIS-C following mRNA vaccines were reported [6].

The MIS-C pathogenesis is still unclear, but it is suggested that it might result from immune dysregulation. Fever is the dominant clinical symptom, followed by mucocutaneous, respiratory, cardiological, gastroenterological, and neurological manifestations [3,4,5]. Laboratory findings reveal significantly increased inflammatory markers. The non-specificity of clinical and laboratory findings poses a substantial diagnostic challenge, but the link to COVID-19 infection is the crucial diagnostic factor for distinguishing MIS-C from other diseases [4,5]. In May 2020, the following three institutions, the World Health Organization (WHO), the Centers for Disease Control and Prevention (CDC), and the Royal College of Pediatrics and Child Health established the case definitions of MIS-C [7,8]. The severity of the clinical presentation requires prompt diagnosis and administration of adequate therapy according to protocols recommended by the American College of Rheumatology (ACR) [4,5]. High-dose intravenous immunoglobulins (IVIGs) and methylprednisolone are considered first-line therapy, while pulse doses of methylprednisolone and biologic agents (anakinra and infliximab) are administered in patients with refractory disease [4,5].

Since the epidemic began, several SARS-CoV-2 variants have been identified, including Wuhan, Alpha, Delta, and Omicron [9]. So far, only a few researchers have offered the analysis of clinical and laboratory features of MIS-C in different periods defined according to the predominant SARS-CoV-2 variant. The results of individual studies suggested that MIS-C characteristics vary according to the SARS-CoV-2 variant [10]. Contrary to that, some other studies did not establish any differences [11,12].

This study aimed to analyze and compare the demographic, clinical, and laboratory characteristics as well as the administered therapy, course, and outcome of the disease in children diagnosed with MIS-C in the territory of the Autonomous Province of Vojvodina according to the predominant SARS-CoV-2 variant.

## 2. Materials and Methods

### 2.1. Study Design an Setting

This retrospective observational study encompassed all patients aged 0–18 diagnosed with MIS-C and treated at the Institute for Child and Youth Health Care of Vojvodina from July 2020 to December 2022 and monitored over a subsequent one-year period after discharge from the hospital.

The study was approved by the Institute’s Ethics Committee and was conducted in accordance with the principles of the Declaration of Helsinki.

The methodology and data obtained in the study were reported, as much as it was feasible, as recommended by the Strengthening the Reporting of Observational Studies in Epidemiology (STROBE) guidelines [13].

### 2.2. Participants

All patients aged 0–18 diagnosed with MIS-C during the observational period mentioned above were included in the study. The diagnosis was established according to the WHO criteria [4]. Exclusion criteria were as follows: incomplete medical documentation, unproven link with SARS-CoV-2 infection, and patient’s withdrawal from follow-up after hospital discharge. Parents or guardians of all participants signed a written informed consent for hospital admission and procedures performed during hospitalization. The patients were distributed according to the predominant SARS-CoV-2 variant in the territory of Province of Vojvodina, as follows: patients hospitalized from the beginning of the pandemic until September 2020—Wuhan period; patients hospitalized from October 2020 to Jun 2021—Alpha period; patients hospitalized from July 2021 to December 2021—Delta period; patients hospitalized from January 2022 to December 2022—Omicron period (periods are classified according to the instructions of the regional Institute for Public Health). The data were obtained from patients’ medical records. A link to SARS-CoV-2 was established according to the positive antigen test to SARS-CoV-2 using oro/nasopharyngeal swabs or positive test to SARS-CoV-2 by reverse transcription polymerase chain reaction (RT-PCR) using oro/nasopharyngeal swabs at admission, positive serology test (IgM and/or IgG for SARS-CoV-2), or confirmed recent SARS-CoV-2 infection in the patient or his/her family members. SARS-CoV-2 IgM and IgG were detected using a chemiluminescence immunoassay (CLIA). The patients included in this study were treated by the protocols recommended by ACR [4,5]. Indications for admission to the PICU were the need for mechanical ventilation and hemodynamic instability.

Patients were monitored over one year after discharge from the hospital, and the outcomes and occurrence of potential complications during the monitoring period were analyzed.

### 2.3. Variables and Data Sources

The data were collected from medical records. Demographic data (age, sex), information on comorbidities, body mass index (BMI), and clinical data (febricity and duration of febrile periods; symptoms of Kawasaki-like phenotypes; presence of respiratory, gastrointestinal, neurological, and other symptoms; hypotension) were collected. The data from the laboratory tests and findings were recorded, taking into account the age-specific reference ranges (blood counts, inflammatory markers, cardiac markers, and markers of renal, liver, and pancreatic function). Procalcitonin (PCT), interleukin (IL)-6, troponin I, N-terminal pro-B-type natriuretic peptide (NT-proBNP), and pancreatic enzymes were not tested in all patients due to technical limitations. The results of chest radiography, lung ultrasonography, echocardiography, and abdominal ultrasound were collected. Also, the data on the administered therapy, need for admission to PICU, and complications occurring during hospitalization were collected, as well as the data on the length of clinical and laboratory recovery.

The variables were compared between the groups of patients distributed according to the presumed SARS-CoV-2 variant (presumption was made on the basis of predominant SARS-CoV-2 variant in the territory of Province of Vojvodina at the time of hospitalization).

### 2.4. Statistical Methods

This study employed a combination of descriptive and inferential statistical methods to analyze the data. In descriptive statistics, median and interquartile ranges (IQR) were used for numerical variables, while frequency and percentage were applied for categorical variables. For the inferential statistics, Kruskal–Wallis ANOVA was used for the comparative analysis of numerical variables. This non-parametric method is appropriate when the assumptions of normality are not met, and it tests whether samples originate from the same distribution. Fisher’s exact test, which was employed for the comparative analysis of categorical variables, is particularly suitable for small sample sizes to determine nonrandom associations between categorical variables. A *p*-value of <0.05 was considered statistically significant. Statistical analyses were performed using Statistica 13.0, a statistical software package (TIBCO Software Inc., Palo Alto, CA, USA), and VassarStats, an online statistical calculator (http://vassarstats.net/ (accessed on 3 November 2024)), on a Windows operating system.

## 3. Results

### 3.1. Participants

**Figure 1:**
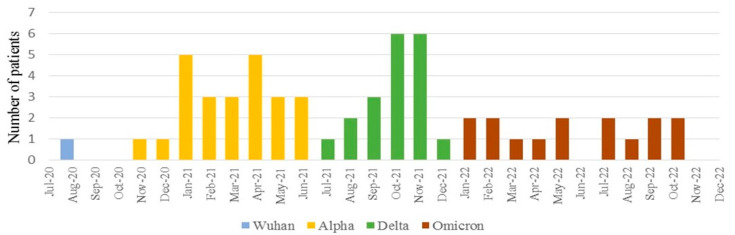
Number of registered MIS-C cases over time in relation to SARS-CoV-2 variant periods.

Of 59 patients diagnosed with MIS-C, regarding the SARS-CoV-2 variant periods, only 1 (2%) case was diagnosed during the Wuhan period, whereas 24 (41%), 19 (32%), and 15 (25%) were diagnosed during the Alpha, Delta, and Omicron periods, respectively (Figure 1). The youngest and oldest patients, aged 4 months and 17 years 8 months, were from the Delta period. There were no statistically significant differences between the three groups of patients with respect to age or sex (Table 1).

Since only one MIS-C case was registered during the Wuhan period, further statistical analysis included only the patients clinically diagnosed with MIS-C during the Alpha, Delta, and Omicron periods. Demographic and clinical characteristics of MIS-C patients according to SARS-CoV-2 variant periods are displayed in Table 1.

### 3.2. Clinical Data

The main clinical characteristics are shown in Table 1. Out of all the patients, 49.09% had comorbidities: overweight and obesity (17.2%), Hashimoto’s thyroiditis (8.6%), inherited cardiac disease (8.6%), undernourishment (6.9%), asthma (5.1%), and individual cases of hypertension, epilepsy, autism, and inherited urinary disorder. There were no statistically significant differences with respect to the comorbidity rate between the three analyzed groups (*p* = 0.79). Multiple comorbidities were identified in two patients from the Alpha period (one patient had three and one had two comorbidities), one patient from the Delta period (two comorbidities), and one patient from the Omicron period (three comorbidities).

Preceding SARS-CoV-2 infection in family members was established in 37.5% of patients from the Alpha period, 10.5% from the Delta period, and 13.3% from Omicron period (*p* = 0.10). Confirmed SARS-CoV-2 infection in a patient’s previous history was recorded in 41.7%, 52.6%, and 20% of patients from the Alpha, Delta, and Omicron periods, respectively (*p* = 0.17). There were no statistically significant differences between the three groups with respect to the period from the previous infection or confirmed SARS-CoV-2 exposure until the occurrence of MIS-C symptoms (*p* = 0.20).

The most prevalent respiratory symptom was cough (29.2% of patients from the Alpha period, 42.1% from the Delta period, and 26.7% from the Omicron period, *p* = 0.63), followed by throat pain (16.7% of patients from the Alpha period, 15.8% from the Delta period, and 6.6% from the Omicron period, *p* = 0.71) and nose congestion (21% of patients from the Delta period and 20% from the Omicron period, *p* = 0.029). Shortness of breath was the most rarely diagnosed respiratory symptom (4.2% of patients from the Alpha period and 5.3% from the Delta period, *p* = 0.99). The most prevalent gastrointestinal symptoms included vomiting and nausea (60.5% of patients from the Alpha period, 52.6% from the Delta period, and 46.7% from the Omicron period, *p* = 0.75), followed by diarrhea (45.8% of patients from the Alpha period, 42.1% from the Delta period, and 33.3% from the Omicron period, *p* = 0.75) and abdominal pain (41.71% of patients from the Alpha period, 15.8% from the Delta period, and 20% from the Omicron period, *p* = 0.16). Headache was the dominant neurological symptom (16.6% of patients from the Alpha period, 15.7% from the Delta period, and 26.6% from the Omicron period). One patient from the Alpha period and one from the Delta period manifested meningism and lapses of consciousness, respectively. Other symptoms identified in the investigated patient population included periungual desquamation in two patients, smell and taste loss in one patient, and testicular swelling in one patient from the Alpha period, pronounced myalgia in two patients and coxalgia in one patient from the Delta period, and highly pronounced fatigue in two patients from the Omicron period.

### 3.3. Laboratory and Radiological Data

Laboratory, radiological, and ultrasound results in MIS-C patients according to SARS-CoV-2 variant periods are displayed in Table 2.

### 3.4. Laboratory Data

Due to technical issues, PCT was performed in only 32 patients, revealing increased values in 83.3% of patients from the Alpha period, 88.9% from the Delta period, and all patients tested during the Omicron period. NT-proBNP was performed in only some patients because of technical limitations, and extremely high values were recorded in patients with cardiovascular involvement. The incidence of abnormal values of serum immunoglobulin and immunology findings has also been investigated. An increased IgM level was observed in one patient from the Alpha and three patients from the Delta periods, while a decreased IgM level was recorded in one patient from the Alpha period. Elevated IgG level was identified in three patients from the Delta and one from the Omicron period, and decreased IgG in seven patients from the Alpha, one from the Delta, and two from the Omicron periods. Immunology tests were performed on a total of 25 patients. Positive antinuclear antibodies were detected in four patients from the Alpha period and three from the Delta period. Positive lupus anticoagulant was found in one patient from the Omicron period, and positive anticardiolipin antibodies were found in one patient from the Delta and one from the Omicron period. Positive beta 2-glycoprotein antibodies were found in one patient from the Omicron period.

### 3.5. Radiography and Imaging Data

The incidence of abnormal chest X-ray findings is shown in Table 1. Forty-nine patients underwent lung ultrasound. Abnormal findings of the lung ultrasound most frequently showed pleural effusion (44.8%), lung condensation (8.1%), and interstitial edema (2%) and were recorded in 52.6% of patients from the Alpha period, 47.4% from the Delta period, and 72.7% from the Omicron period (*p* = 0.29). Magnetic resonance imaging of the endocranium was performed in one child from the Omicron period, revealing vasculitis.

Therapy, complications, and outcomes in MIS-C patients according to SARS-CoV-2 variant periods were presented in Table 3.

### 3.6. Therapy

All patients were administered IVIG doses at 2 g/kg. Methylprednisolone was administered in 94.8% of patients at 1–2 mg/kg. There were no statistically significant differences between the three investigated groups with respect to either administered methylprednisolone doses (Alpha period 1 [1–2], Delta period 1 [1–2], Omicron period 1 [1–1], *p* = 0.39) or duration of corticosteroid therapy (weeks: Alpha period 4 [3–4], Delta period 4 [3–4], Omicron period 4 [4–4], *p* = 0.54). Methylprednisolone pulses were applied in two patients at 30 mg/kg doses. Acetylsalicylic acid was administered in 98.3% of patients at an anti-aggregation dose of 3–5 mg/kg. Low molecular heparin was administered to 52 (88.13%) patients as a first-line treatment, before initiation of acetylsalicylic acid. There were no statistically significant differences between the three groups with respect to either administered acetylsalicylic acid dose (Alpha period 3 [3–5], Delta period 4 [3–5], Omicron period 3 [3–5], *p* = 0.53) or the duration of acetylsalicylic acid therapy (weeks: Alpha period 6 [6–6], Delta period 6 [4–6], Omicron period 4 [4–6], *p* = 0.50).

### 3.7. Acute Complications

In all three groups, acute pancreatitis was the most common complication during hospitalization (*p* = 0.72). A total of 8.6% of patients were hospitalized in the pediatric intensive care unit, and mechanical ventilation was required in two patients (one from the Delta and one from the Omicron period). The differences between the three groups regarding clinical and laboratory recovery periods were not statistically significant.

### 3.8. Long-Term Outcomes

In total, 94.8% of patients had favorable outcomes with no complications, while one patient from the Delta period, who was extremely obese, died due to a pulmonary embolism. We had two patients with chronic complications, one patient with pancreatitis (Delta period), and one patient with proteinuria (Omicron period). At the end of the one-year follow-up period, the patient with pancreatitis had a complete resolution of symptoms and normalization of laboratory findings. In the one patient with proteinuria, the laboratory findings were maintained after one year, but at the level of insignificant proteinuria (24 h proteinuria: 0.17 g/dU, 24 h microalbuminuria 30.8 mg/dU, dU 1940 mL).

## 4. Discussion

During the SARS-CoV-2 pandemic, virus mutations affected the changes in the clinical picture of COVID-19. The question was raised about whether virus mutation could influence the expression of delayed post-infectious complications of COVID-19 infection, such as MIS-C. This study analyzed and compared demographic, clinical, and laboratory features, administered therapy, disease course, and outcome in children diagnosed with MIS-C in the territory of Vojvodina Province concerning the predominant SARS-CoV-2 variant. The investigation included 59 patients diagnosed with MIS-C (1 patient from the Wuhan period, 41% from the Alpha period, 32% from the Delta period, and 25% from the Omicron period). Exceptionally low MIS-C incidence during the Wuhan period could be attributed to the inconsistent diagnostic procedures and low incidence of COVID-19 in this period due to a lockdown of children communities and curfew measures in the Republic of Serbia, and hence Vojvodina Province as a part thereof. The study revealed no statistically significant differences in the distribution of patients from the Alpha, Delta, and Omicron periods according to age, corresponding with the results reported in most other studies [10,11,14]. However, a multicentric study by Sperotto F et al. reported a significantly younger age of patients from the Delta and the Omicron periods compared to the Alpha period; a possible explanation for that result may be a potential predisposition of younger-aged patients to new SARS-CoV-2 variants, differences in immune response to different virus variants, and vaccinal status [15]. The results of numerous types of research indicated the predomination of male patients in MIS-C, which might be attributed to the hormonal or genetic (i.e., X chromosome) differences and possible effects of the angiotensin-converting enzyme (ACE-2) receptor expression [16]. Our study also confirmed the domination of male patients in both the whole cohort and all investigated groups; however, the differences according to sex structure were not statistically significant. The comorbidities, particularly overweight and asthma, are potential risk factors for MIS-C development [17,18]. In our study, comorbidities were recorded in about half of the patients, overweight and obesity being the most common ones. There were no statistically significant differences in comorbidities between patients from the Alpha, Delta, and Omicron periods, and similar results were reported by other authors [10,12,15]. Confirmed SARS-CoV-2 infection in the patient’s history was registered in less than one-half of patients, which might be associated with a commonly mild clinical presentation or an asymptomatic course of COVID-19 infection in children, and SARS-CoV-2 testing is typically not performed in children with mild infections. Positive PCR to SARS-CoV-2 was recorded in a small number of patients from Alpha and Delta periods, which could be explained by a shorter post-infection persistence of the Omicron-variant viral genome [19]. SARS-CoV-2 positive IgG was identified in more than half of our patients from the Alpha and the Delta periods and in all patients from the Omicron period. During the pandemic, serological methods for diagnosing preceding SARS-CoV-2 infection have significantly improved and become more sophisticated. However, considering the high virus seroprevalence in the population, suspected MIS-C in the case of positive IgG is the only evidence of preceding COVID-19 infection, inevitably requiring comprehensive differential diagnostics [10,20].

The spectrum of MIS-C clinical symptoms and signs is broad, and the severity of individual symptoms and signs dramatically varies. High body temperature is a consistent symptom recorded in all patients participating in our study; there were no significant differences in the febricity periods between the three groups. Furthermore, there were no significant differences in the prevalence of individual clinical signs of Kawasaki-like phenotype between the groups, which corresponds with the results reported by other authors [5,10,15,21]. According to one of the most comprehensive wide-scale meta-analyses involving almost 4500 MIS-C cases, the most common clinical symptoms following febricity were gastrointestinal (52%). In contrast, respiratory and neurological symptoms were recorded in 42% and 27% of patients, respectively [22]. In our study, 80% of patients manifested gastrointestinal symptoms, and the incidence of respiratory and neurological symptoms was similar to that reported in the meta-analysis mentioned above. There were no significant differences in the prevalence of gastrointestinal and neurological symptoms between the three investigated groups, which corresponds with the results of previous studies [6,23,24]. However, the incidence of respiratory symptoms recorded in our research was considerably higher in patients from the Delta period than in those from the Alpha and the Omicron period. Literature data on the prevalence of respiratory symptoms according to the predominant SARS-CoV-2 variant are somewhat inconsistent. The study by Ptak K et al., which included 108 MIS-C cases, revealed a significantly higher incidence of respiratory symptoms in patients from the Delta/Omicron periods compared to those from the Alpha period [12]. The research by Sperotto F. et al. encompassed 588 MIS-C cases; a significantly higher incidence of respiratory symptoms was recorded in the Alpha period than in the Delta and the Omicron periods [15], whereas some authors did not report any differences in the incidence of respiratory symptoms according to the SARS-CoV-2 variant [6,10]. The data discrepancy might be due to the inconsistency in cohort characteristics between the studies and the non-uniformity in defining the respiratory symptoms.

Typical laboratory findings in MIS-C include lymphopenia, neutrophilia, thrombocytopenia, moderate anemia, abnormal pathological values of inflammatory markers such as ESR, CRP, fibrinogen, ferritin, PCT, and IL-6, and elevated LDH levels, hypoalbuminemia, and hyponatremia. Coagulation mechanism alteration (elevated D-dimer, prolongation of both PT and aPTT) occurs frequently, and cardiac enzymes, such as troponin and NT-pro BNP, are commonly crucial for establishing the diagnosis [16,22]. CRP values, neutrophilia, lymphopenia, and hypoalbuminemia correlated with disease severity [16,25]. Our study did not reveal any statistically significant differences in laboratory markers of inflammation or visceral organ lesions between patients from the Alpha, Delta, and Omicron periods. The results reported by other authors are inconsistent, but some studies also could not establish significant differences in laboratory findings according to the SARS-CoV-2 variant [6,12,21]. The importance of the results obtained in our study is limited by technical limitations for determining specific laboratory parameters (such as PCT and cardiac enzyme) in all patients.

All patients from our study underwent chest radiography, and there were no significant differences in the incidence of abnormal chest radiographs between the groups. Lung ultrasonography was performed in most patients; the method proved to be more sensitive for detecting pathological changes in the lungs than radiography. Cardiac involvement is a potentially highly severe MIS-C complication, which can manifest as myocarditis, ventricular dysfunction, valvular regurgitation, pericarditis, arrhythmia, or coronary artery abnormalities (CAAs). Myocarditis occurs more frequently in older children, whereas the risk from CAA development is higher in younger children [26,27]. Pericarditis is considered a less common cardiac involvement; however, in our study, pericarditis was the most commonly detected abnormality in the echocardiography. Cardiac involvement was identified in less than one-third of our patients, without statistically significant differences in incidence between patients from the Alpha, Delta, and Omicron periods. Contrary to that, the study by Khan R et al. reported cardiac involvement in 87% of patients and the differences in manifestations according to the predominant SARS-CoV-2 variant with the most severe cardiac involvement in patients from the Delta period [14].

MIS-C and Kawasaki disease (KD) are considered two distinctive diseases triggered by different infectious agents. These two entities share many of the same clinical and laboratory characteristics [28]. Differentiation between MIS-C and KD can cause difficulties due to their many similarities, especially in children under 5 years of age. Those diseases may belong to the same spectrum of inflammatory disorders but differ in many aspects of etiology, demography, epidemiology, clinical and laboratory findings, and pathology. As stated in the article by Rivas et al., the intensity of the inflammatory response and long-term cardiovascular sequelae diverge between KD and MIS-C. On the other hand, there is obvious diversity between the two illnesses, as there are signs and symptoms with high frequency in MIS-C which are less commonly or rarely presented in KD patients [29]. MIS-C presents as a more intense inflammatory syndrome, myocardial dysfunction, and cardiogenic shock, whereas KD is typically associated with changes in the coronary arteries [28]. For the purpose of our study, patients were diagnosed as MIS-C if they did not fulfill all the clinical criteria of KD, had a clear link with previous COVID-19 infection, and had cardiac involvement (LV dysfunction, myo/pericarditis, cardiogenic shock, arrhythmia, etc.) other than coronary aneurysm. So, in our cohort, there were no patients who fulfilled the criteria for KD.

All the patients included in our study were treated using the protocols recommended by ACR [4,5], and there were no significant differences in MIS-C therapies throughout the variant periods. Methylprednisolone pulse was administered in only 3.4% of patients, and considering a favorable response to initial therapy, biologics were not used in any of the patients. Prompt MIS-C diagnosis and timely adequate treatment are the prerequisites for a favorable disease outcome. Complications during hospitalization occurred in 36.2% of our patients, and admission to PICU was indicated in 8.6%. We did not establish any significant differences in the incidence of complications during hospitalization and the number of patients hospitalized in PICU between patients from the Alpha, Delta, and Omicron periods. Literature data on the number of patients with MIS-C hospitalized in PICUs significantly vary, and the differences result not only from the severity of the clinical picture but also from the local guidelines for indications for PICU admission. In our patients, indications for admission to PICU included the need for mechanical ventilation and hemodynamic instability. The outcome of MIS-C in children is most commonly favorable [17]. In our study, the patients were monitored over a one-year period. The outcome after hospital discharge was favorable in almost all patients, without significant differences between those from the Alpha, Delta, and Omicron periods. According to the data from the literature, the mortality rate in MIS-C ranges from 1.7 to 3.2%, with an increased risk of fatal outcomes in the adolescent population and patients with comorbidities [16,22,30]. Within our cohort, post-discharge mortality due to a pulmonary embolism occurred in only one patient from the Delta period, who was extremely obese.

### Limitations of the Study

Several limitations exist in the current study. First of all, the number of patients investigated was small. However, our study represents the incidence of MIS-C in the territory of the Autonomous Province of Vojvodina since the Institute for Child and Youth Health Care of Vojvodina-pediatric COVID-19 center is the only institution in Vojvodina Province where pediatric patients with MIS-C were diagnosed and treated, which contributes to the importance of the obtained results. A single-center study has its limitations, but on the other hand, the same team conducted the diagnostics, treatment, and monitoring of all patients by providing uniform diagnostic and therapeutic approaches and consistent patient monitoring. The main limitation was the fact that the COVID variant was not determined. Patients were only grouped according to hospitalization date, making correlations between COVID variant and MIS-C unreliable. However, the study was based on data regarding the dominant virus variant in the territory where patients lived when a diagnosis of MIS-C was established. Some laboratory analyses were not performed in all patients due to technical limitations; however, considering that neither single laboratory finding itself is specific for MIS-C, as well as a broad spectrum of other laboratory tests and diagnostic procedures conducted, we are of the opinion that it has not substantially affected the obtained results. Moreover, our study did not analyze the effects of vaccination on MIS-C expression. This was due to low vaccination rates among children in the territory of Vojvodina Province, and none of the patients from the investigated cohort were vaccinated.

## 5. Conclusions

Even though the clinical expression of COVID-19, especially during the Omicron period, became significantly milder over time, our study did not reveal any evident changes in the presentation and severity of MIS-C. Thus, we may conclude that MIS-C still represents a severe, potentially life-threatening, delayed post-infection COVID-19 complication, which should not be neglected and still requires prompt diagnosis and adequate treatment. Despite the limitations of our study, this investigation contributed to the understanding of MIS-C. Further monitoring of children with MIS-C and additional studies will offer an answer to the question of whether the SARS-CoV-2 variant affects the presentation and severity of MIS-C.

## Figures and Tables

**Table 1:** Demographic and clinical characteristics of MIS-C patients according to SARS-CoV-2 variant periods.

Characteristic	Total n = 58	Alpha n = 24 (41%)	Delta n = 19 (33%)	Omicron n = 15 (26%)	*p* Value
Age (years), median [IQR]	7.8 [2.8–11.0]	9.15 [4.6–11.75]	7.8 [2.8–13]	6.5 [2.3–10.6]	0.34
Female (%) Male (%)	21 (36.2) 37 (63.8)	7 (29.2) 17 (70.8)	9 (47.4) 10 (52.6)	5 (33.3) 10 (66.7)	0.39
Comorbidities, (%)	27 (49.09)	11 (47.83)	10 (55.56)	6 (42.86)	0.79
BMI percentile, median [IQR]	45 [11–84]	67 [24–90]	61 [20–95]	16 [10–40]	0.07
Confirmed SARS-CoV-2 infection in the family, (%)	13 (22.4)	9 (37.5)	2 (10.5)	2 (13.3)	0.10
Confirmed SARS-CoV-2 infection in the patient’s past hystory, (%)	23 (39.7)	10 (41.7)	10 (52.6)	3 (20.0)	0.17
Timing of preceding illness or preceding SARS-CoV-2 exposure (weeks), median [IQR]	4 [2–4]	4 [2–6]	2.5 [2–4]	4 [3–8]	0.20
SARS-CoV-2 at MIS-C diagnosis Antigen test positive, (%) PCR positive, (%) SARS-CoV-2 IgM positive, (%) SARS-CoV-2 IgM (RR < 1.0 BAU/mL) median [IQR] SARS-CoV-2 IgG positive, (%) SARS-CoV-2 IgG (RR < 7.1 BAU/mL) median [IQR]	3 (5.2) 8 (13.8) 4 (6.9) 2.37 [1.97–7.47] 40 (68.9) 145.2 [55.5–51.1]	2 (8.3) 4 (16.7) 0 (0.0) none / 13 (54.2) 309.5 [25.2–645.0]	1 (5.3) 4 (21.0) 3 (15.8) 2.7 [1.9–12.2] 12 (63.1) 168.4 [67.2–341.7]	0 (0.0) 0 (0.0) 1 (6.7) 2.0 [2.0–2.0] 15 (100.0) 109.3 [55.5–451.1]	0.78 0.17 0.12 n.a. **0.003** 0.97
Fever, median [IQR]	39.5 [39.1–40.0]	39.5 [39.2–40.0]	39.5 [39.0–39.9]	39.5 [39.0–40.0]	0.82
Duration of fever (days), median [IQR]	6 [5–8]	7 [5–8]	8 [5–9]	6 [3–7]	0.60
Rash, (%)	24 (41.4)	10 (41.7)	8 (42.1)	6 (40.0)	0.99
Bilateral bulbar conjunctival injection, (%)	20 (34.5)	11 (45.8)	5 (26.3)	4 (26.7)	0.34
Changes in lips/oral cavity, (%)	19 (32.7)	10 (41.7)	4 (21.0)	5 (33.3)	0.33
Cervical lymphadenopathy (>1.5 cm), (%)	25 (43.1)	9 (37.5)	7 (36.8)	9 (60.0)	0.33
Edema of hands/feet, (%)	8 (13.8)	4 (16.7)	3 (15.8)	1 (6.7)	0.71
Erythema of palms/soles, (%)	7 (12.1)	4 (16.7)	1 (5.3)	2 (13.4)	0.52
Respiratory symptoms, (%)	28 (48.3)	9 (37.5)	14 (73.7)	5 (33.3)	**0.028**
Gastrointestinal symptoms, (%)	47 (81.0)	21 (87.5)	16 (82.2)	10 (66.7)	0.30
Neurologycal symptoms, (%)	13 (22.4)	5 (20.8)	4 (21.0)	4 (26.7)	0.85
Hypotension *, (%)	4 (6.9)	1 (4.2)	1 (5.3)	2 (13.4)	0.67
Other symptoms, (%)	9 (15.5)	4 (16.7)	3 (15.8)	2 (13.4)	0.99

IQR: interquartile range; f: frequency; RR: *reference range;* BMI: body mass index; SARS-CoV-2: severe acute respiratory syndrome coronavirus 2; MIS-C: multisystem inflammatory syndrome in children; PCR: polymerase chain reaction; Ig: immunoglobulin; * Hypotension: systolic blood pressure <5th centile for age-specific values.

**Table 2:** Laboratory, radiological, and ultrasound findings in MIS-C patients according to SARS-CoV-2 variant periods.

Findings	Total n = 58	Alpha n = 24 (41%)	Delta n = 19 (33%)	Omicron n = 15 (26%)	*p* Value
Erytrocyte sedimentation (mm/h), median [IQR]	65 [47–75]	59 [48.5–87.0]	68 [52–75]	54 [30–68]	0.34
Leukocytes (RR 5–10 × 10^9^/L), median [IQR] Leukocytosis, (%) Leukopenia, (%)	11.1 [6.95–17.50] 32 (55.2) 2 (3.4)	10.2 [6.95–14.30] 10 (41.7) 1 (4.2)	15.2 [9.8–21.4] 13 (68.4) 1 (5.3)	11.3 [8.1–17.7] 9 (60.0) 0 (0.0)	0.16 0.21 0.99
Neutrophils (RR 2.5–8.5 × 10^9^/L), median [IQR] Neutrophilia, (%)	9.15 [5.5–13.39] 37 (63.8)	8.50 [5.04–11.79] 14 (58.3)	12.2 [7.17–15.27] 15 (78.9)	9.15 [5.4–13.14] 9 (60.0)	0.15 0.32
Lymphocytes (1.3–4.5 × 10^9^/L), median [IQR] Lymphopenia, (%)	1.33 [0.82–2.21] 18 (31.0)	1.30 [0.81–1.93] 8 (33.3)	1.79 [0.76–3.43] 7 (36.8)	1.45 [1–2.21] 3 (20.0)	0.48 0.39
Hemoglobin (RR 100–140 g/L), median [IQR] Anemia, (%)	105 [94–120] 39 (67.2)	103.5 [94–120.5] 16 (66.7)	108 [97–120] 14 (73.7)	103 [89–120] 9 (60.0)	0.82 0.68
Platelets (RR 150–400 × 10^9^/L), median [IQR] Thrombocytosis, (%) Thrombocytopenia, (%)	250 [202–364] 9 (15.5) 0 (0.0)	258.5 [200–296] 2 (8.3) 0 (0.0)	300 [222–410] 3 (15.8) 0 (0.0)	210 [184–509] 4 (26.7) 0 (0.0)	0.33 0.35
C-reactive protein (RR < 5 mg/L), median [IQR] Raised CRP, (%)	163 [106–249.9] 58 (100)	190 [129.4–249.3] 24 (100)	160 [76.6–249] 19 (100.0)	156 [63–262] 15 (100.0)	0.54 0.99
Fibrinogen (RR 1.86–4 g//L), median [IQR] Raised fibrinogen, (%)	5.61 [4.80–7.58] 56 (96.6)	5.60 [4.94–7.15] 23 (95.8)	5.47 [4.46–7.68] 18 (94.7)	5.9 [5.36–7.71] 15 (100.0)	0.64 0.99
Feritin (RR 22–275 ug/L), median [IQR] Raised feritin, (%)	438 [284–715] 45 (77.6)	345 [230–637.5] 18 (75.0)	500 [240–879] 13 (68.4)	468 [290–979] 14 (93.3)	0.53 0.20
Prokalcitonin (RR < 0.05 ng/mL), median [IQR] Raised PCT, (%)	n = 32 2.57 [0.89–8.41] 29 (90.6)	n = 12 3.64 [0.92–11.08] 10 (83.3)	n = 9 0.98 [0.61–6.19] 8 (88.9)	n = 11 3.19 [2.43–17.9] 11 (100.0)	0.32 0.48
Interleukin-6 (RR < 7 pg/mL), median [IQR] Raised IL-6, (%)	n = 55 83 [35.1–384] 55 (100.0)	n = 22 66.3 [39.8–338.2] 22 (100.0)	n = 19 81.0 [31.9–360] 19 (100.0)	n = 14 145.25 [56.4–725] 14 (100.0)	0.34 0.99
Troponin I (RR < 25 ng/L), median [IQR] Raised troponin I, (%)	n = 44 25.6 [6.25–55.6] 29 (65.9)	n = 19 36.0 [5.9–69.8] 14 (73.7)	n = 14 11.5 [5.4–24.5] 6 (42.7)	n = 11 36.8 [21–502.9] 9 (81.8)	0.057 0.18
NT-proBNP (RR < 125 pg/mL), median [IQR] Raised NT-proBNP, (%)	n = 9 394 [7–5623] 5 (55.6)	n = 1 7 [7–7] 0 (0.0)	n = 3 9.1 [3.47–622] 1 (33.3)	n = 5 5623 [394–20,409] 4 (80.0)	0.45 0.29
Aspartate aminotransferase (RR 0.08–0.6 ukat/L), median [IQR] Raised AST, (%)	0.67 [0.39–1.09] 29 (50.0)	0.79 [0.42–1.10] 13 (54.2)	0.45 [0.38–1.01] 7 (36.8)	0.72 [0.42–2.13] 9 (60.0)	0.29 0.38
Alanine aminotransferase (RR 0.20–0.98 ukat/L), median [IQR] Raised ALT, (%)	0.47 [0.26–1.15] 23 (39.7)	0.49 [0.30–1.04] 10 (41.7)	0.47 [0.25–1.06] 7 (36.8)	0.34 [0.25–1.97] 6 (40.0)	0.88 0.94
Lactate dehydrogenase (RR 1.83–4.92 ukat/L), median [IQR] Raised LDH, (%)	4.58 [3.50–5.39] 25 (43.1)	4.57 [3.59–5.01] 9 (37.5)	4.96 [3.43–5.99] 10 (52.6)	4.4 [3.6–5.91] 6 (40.0)	0.84 0.56
Gamma-glutamyl transferase (RR 0.07–0.37 ukat/L) median [IQR] Raised GGT, (%)	0.55 [0.25–1.20] 38 (65.5)	0.62 [0.24–1.55] 16 (66.7)	0.39 [0.23–1.11] 11 (57.9)	0.48 [0.43–1.2] 11 (73.3)	0.83 0.65
Urea (RR 2.5–6 mmol/L), median [IQR] Raised urea, (%)	3.7 [2.7–5.0] 6 (10.3)	3.7 [2.9–5.4] 5 (20.8)	3.4 [2.2–4.9] 0 (0.0)	4.4 [3.6–5.6] 1 (6.7)	0.11 0.09
Creatinine (RR 40–68 umol/L), median [IQR] Raised creatinine, (%)	39 [28–50] 6 (10.3)	42.5 [31.0–63.5] 5 (20.8)	38.3 [23.5–48] 0 (0.0)	37.3 [26–48] 1 (6.7)	0.45 0.09
Uric acid (RR 143–339 umol/L), median [IQR] Raised uric acid, (%)	221 [163–270] 6 (10.3)	223 [166.4–284.0] 5 (20.8)	218 [152–269] 0 (0.0)	236 [159–281] 1 (6.7)	0.74 0.09
Albumin (RR 38–54 g/L), median [IQR] Hypoalbuminemia, (%)	28.1 [26.6–33.0] 52 (89.7)	29.1 [26.8–32.5] 20 (83.3)	28.7 [26.4–34.0] 17 (89.5)	27.7 [26.6–29.4] 15 (100.0)	0.45 0.26
Sodium (RR 135–145 mmol/L), median [IQR] Hyponatremia, (%)	133 [130–137] 37 (63.8)	133 [130–137] 15 (62.5)	132 [129–135] 11 (57.9)	132 [129–135] 11 (73.3)	0.64 0.69
Lipase (RR 0.07–0.65 ukat/L) median [IQR] Raised lipase, (%)	n = 18 2.02 [1.32–3.89] 16 (88.9)	n = 6 2.08 [0.80–3.97] 6 (100.0)	n = 7 3.29 [1.32–3.89] 5 (71.4)	n = 5 1.97 [1.35–3.58] 5 (100.0)	0.98 0.65
Amylase (RR 0.46–1.66 ukat/L) median [IQR] Raised amylase, (%)	n = 18 2.84 [1.7–3.48] 14 (77.8)	n = 6 2.68 [1.70–3.48] 5 (83.3)	n = 7 2.84 [1.30–3.09] 5 (71.4)	n = 5 3.15 [2.39–3.81] 4 (80.0)	0.76 0.99
PT (RR 10–13,1 sec), median [IQR] Prolonged PT, (%)	14.6 [13.1–17.3] 43 (74.1)	14.5 [13.1–17.5] 17 (70.8)	14.7 [13.9–17.2] 16 (84.2)	13.9 [12–17.5] 10 (66.7)	0.66 0.55
aPTT (RR 24–35 sec), median [IQR] Prolonged aPTT, (%)	35 [31.3–42.2] 25 (43.1)	36.2 [33.0–40.5] 12 (50.0)	35 [30.1–39.3] 6 (31.6)	35.1 [31.2–45.2] 7 (46.7)	0.62 0.50
D-dimer (RR <230 ng/mL) median [IQR] Raised D-dimer, (%)	n = 57 1622 [915–2500] 56 (98.2)	n = 23 1625 [945–2500] 22 (95.6)	n = 19 1235 [845–2500] 19 (100.0)	n = 15 2190 [915–2500] 15 (100.0)	0.46 0.33
Abnormal chest radiography, (%) Consolidations (%) Mild pleural effusion, (%)	25 (43.1) 17 (29.3) 8 (13.8)	12 (50.0) 7 (29.2) 5 (20.8)	8 (42.1) 6 (31.6) 2 (10.5)	5 (33.3) 4 (26.7) 1 (6.7)	0.67
Abnormal echocardiogram, (%) Pericarditis, (%) Myopericarditis, (%) Mitral valve regurgitation, (%) Tricuspid valve regurgitation, (%) Heart failure, (%)	17 (29.3) 8 (13.8) 1 (1.7) 3 (5.2) 2 (3.4) 3 (5.2)	9 (37.5) 4 (16.7) 1 (4.2) 2 (8.3) 2 (8.3) 0 (0.0)	2 (10.5) 1 (5.3) 0 (0.0) 0 (0.0) 0 (0.0) 1 (5.3)	6 (40.0) 3 (20.0) 0 (0.0) 1 (6.7) 0 (0.0) 2 (13.3)	0.097
Abnormal abdominal US, (%) Splenomegaly, (%) Bowel wall thickening, (%) Ascites, (%) Mesenteric lymphadenitis, (%) Invagination, (%)	38 (65.5) 20 (34.5) 10 (17.2) 7 (12.0) 4 (6.9) 1 (1.7)	17 (70.83) 9 (37.5) 5 (20.8) 5 (20.8) 0 (0.0) 0 (0.0)	12 (63.16) 7 (36.8) 3 (15.8) 0 (0.0) 1 (5.3) 0 (0.0)	9 (60.00) 4 (26.7) 2 (13.3) 2 (13.3) 3 (20.0) 1 (6.7)	0.78

IQR: interquartile range; RR: reference range; *CRP*: C-reactive protein; PCT: procalcitonin; IL: interleukin; NT-proBNP: N-terminal pro B-type natriuretic peptide; AST: aspartate aminotransferase; ALT: alanine aminotransferase; LDH: lactate dehydrogenase; GGT: gamma-glutamyl transferase; PT: prothrombin time; aPTT: activated partial thromboplastin time; US: ultrasonography.

**Table 3:** Therapy, complications, and outcome in MIS-C patients according to SARS-CoV-2 variant periods.

Variable		Total n = 58	Alpha n = 24 (41%)	Delta n = 19 (33%)	Omicron n = 15 (26%)	*p* Value
Pharmacotherapy	IVIG, (%) Methylprednisolone, (%) Methylprednisolone pulse, (%) Inotrope, (%) Acetylsalicylic acid, (%)	58 (100.0) 55 (94.8) 2 (3.4) 3 (5.2) 57 (98.3)	24 (100.0) 23 (95.8) 0 (0.0) 1 (4.2) 24 (100)	19 (100.0) 18 (94.7) 1 (5.3) 1 (5.3) 19 (100)	15 (100.0) 14 (93.3) 1 (6.7) 1 (6.7) 14 (93.3)	0.99 0.99 0.31 0.99 0.26
Complications During Hospitalization, (%) Pancreatitis, (%) Surgery due to suspected acute abdomen, (%) Acute kidney injury, (%) Heart failure, (%) Acute kidney injury and pancreatitis, (%) Need for mechanical ventilation, (%) Pulmonary edema, (%) Varicella, (%)		21 (36.2) 12 (20.7) 3 (5.2) 3 (5.2) 3 (5.2) 2 (3.5) 2 (3.5) 1 (1.7) 1 (1.7)	10 (41.7) 4 (16.7) 3 (12.5) 2 (8.3) 0 (0.0) 1 (4.2) 0 (0.0) 0 (0.0) 0 (0.0)	5 (26.3) 4 (21.1) 0 (0.0) 0 (0.0) 1 (5.3) 1 (5.3) 1 (5.3) 0 (0.0) 0 (0.0)	6 (40.0) 4 (26.7) 0 (0.0) 1 (6.7) 2 (13.3) 0 (0.0) 1 (6.7) 1 (6.7) 1 (6.7)	0.58
PICU admission, (%)		5 (8.6)	1 (4.2)	2 (10.5)	2 (13.3)	0.81
Duration of clinical recovery after starting therapy (days), median [IQR]		4 [3–6]	4 [3–5.5]	4 [3–6]	5 [4–7]	0.18
Duration of laboratory recovery after starting therapy (days), median [IQR]		10 [7–11]	10 [8–12]	10 [6–11]	9 [7–10]	0.81
Outcomes after hospital discharge	Favorable Outcome, (%) Death, (%) Chronic pancreatitis, (%) Appearance of proteinuria, (%)	55 (94.8) 1 (1.7) 1 (1.7) 1 (1.7)	24 (100.0) 0 (0.0) 0 (0.0) 0 (0.0)	17 (89.5) 1 (5.3) 1 (5.3) 0 (0.0)	14 (93.3) 0 (0.0) 0 (0.0) 1 (6.7)	0.34

IVIG: intravenous immunoglobulin; PICU: pediatric intensive care unit; f: frequency.

## Data Availability

The data presented can be provided on request.
